# Minimal invasive versus open esophagectomy for patients with esophageal squamous cell carcinoma after neoadjuvant treatments

**DOI:** 10.1186/s12885-021-07867-9

**Published:** 2021-02-09

**Authors:** Dongni Chen, Weidong Wang, Junxian Mo, Qiannan Ren, Huikai Miao, Youfang Chen, Zhesheng Wen

**Affiliations:** 1 Department of Thoracic Oncology, State Key Laboratory of Oncology in South China, Collaborative Innovation Center for Cancer Medicine, Sun Yat-sen University Cancer Center, 651 Dongfengdong, Guangzhou, Guangdong 510060 P. R. China; 2 Department of Thoracic Surgery, The First Affiliated Hospital, School of Medicine, Zhejiang University, Hangzhou Zhejiang, 310003 P. R. China; 3 Department of Cardio-Thoracic Surgery, The Seventh Affiliated Hospital of Guangxi Medical University, Wuzhou, 543000 Guangxi China; 4 Department of Experimental Research, State Key Laboratory of Oncology in South China, Collaborative Innovation Center for Cancer Medicine, Sun Yat-sen University Cancer Center, Guangzhou, 510060 P. R. China

**Keywords:** Esophageal squamous cell carcinoma, Neoadjuvant treatment, Minimally invasive esophagectomy, Open surgery

## Abstract

**Background:**

Although previous studies have discussed whether the minimally invasive esophagectomy (MIE) is superior to open surgery, the data concerning esophageal squamous cell carcinoma (ESCC) patients underwent neoadjuvant treatment followed by radical resection is limited. The purpose of our study was to compare the short- and long-term clinical outcomes of the two surgical approaches in treating ESCC patients.

**Methods:**

Between January 2010 and December 2016, ESCC patients who had received neoadjuvant therapy and underwent Mckeown esophagectomy at our institute were eligible. The baseline characteristics, pathological data, short-and long-term outcomes of these patients were collected and compared based on the surgical approach.

**Results:**

A total of 195 patients was included in the current study. Compared to patients underwent open surgery, patients underwent MIE had shorter operative time and less intraoperative bleeding (390 min vs 330 min, *P* = 0.001; 204 ml vs 167 ml, *P* = 0.021). In addition, the risk of anastomotic leakage was decreased in MIE group (20.0% vs 3.3%, *P* < 0.001), while the occurrence of other complications did not have statistical significance between two groups. Overall survival (OS) and disease-free survival (DFS) was no difference in patients received neoadjuvant chemotherapy between the two approaches. For the patients underwent neoadjuvant chemoradiotherapy, OS was significantly better in the MIE group (log rank = 6.197; *P* = 0.013).

**Conclusion:**

Minimally invasive Mckeown esophagectomy is safe and feasible for ESCC patients who underwent neoadjuvant therapy. MIE approach presented better perioperative results than open esophagectomy. The effect of surgical approaches on survival was depending on the scheme of neoadjuvant treatment.

## Background

The burden of esophageal cancer (EC) is expected to rise in China in the coming years, and China nearly accounts for 50% of the world’s EC incidence [1]. The most common esophageal cancer subtype in China is esophageal squamous cell carcinoma (ESCC), affecting predominantly smoking males [1]. Because the early stage of ESCC is usually asymptomatic, most patients are diagnosed with locally advanced disease, where the comprehensive treatment included surgery, radiotherapy and chemotherapy was applied [2]. It is standard to treat locally advanced disease with neoadjuvant chemoradiotherapy (nCRT) and neoadjuvant chemotherapy (nCT) followed by surgery, after the numerous randomized clinical trials have demonstrated the benefits of neoadjuvant therapies for esophageal cancer [3–5]. Compared with those treated with surgery alone, patients treated with nCRT and nCT followed by surgery had a similar postoperative complication rate and perioperative mortality [3]. In addition, neoadjuvant treatment contributes to the shrinkage of the primary tumor and reduction of lymph node metastases, which is a protective prognostic factor.

Esophagectomy with radical lymphadenectomy, usually after nCRT and nCT, is regarded as the best option of multimodality treatment for resectable esophageal cancer [6, 7]. The traditional surgical approach is open esophagectomy, performed through a right thoracotomy and laparotomy. With the development of endoscopic techniques, minimally invasive surgery with video-assisted thoracoscopy are being increasing implemented since its introduction in the 1990s [8, 9]. Minimal invasive esophagectomy (MIE) could reduce the perioperative complications as a result of surgical trauma from open procedures, and MIE patients experience less postoperative pain [10, 11]. Because of these potential advantages, MIE procedures are favored whenever suitable. However, neoadjuvant therapy could result in adverse effect on several organ systems, such as myelotoxicity, cardiac, liver and kidney toxicity. Patients underwent neoadjuvant treatment, who may suffer from thrombocytopenia and potentially increase the risk of bleeding during surgery [12]. In addition, radiation-induced fibrosis contributes to the dissection of the primary tumor and lymph nodes more difficult, making an accidental injury of adjacent structures during resection more likely [13]. In such a condition, open esophagectomy seems to have advantages in surgery because it may be safer and more effective to deal with bleeding and tissue adhesion under direct vision, although it leads to the relatively large incisions. Therefore, the more appropriate surgical procedure for ESCC patients received neoadjuvant therapy is still uncertain. In the current study, to compare MIE and traditional open esophagectomy in terms of short and long-term clinical outcomes, we performed a retrospective study at a single large cancer center, demonstrating the optimal surgical method for ESCC patients underwent neoadjuvant treatment.

## Methods

### Study population

**Fig. 1:**
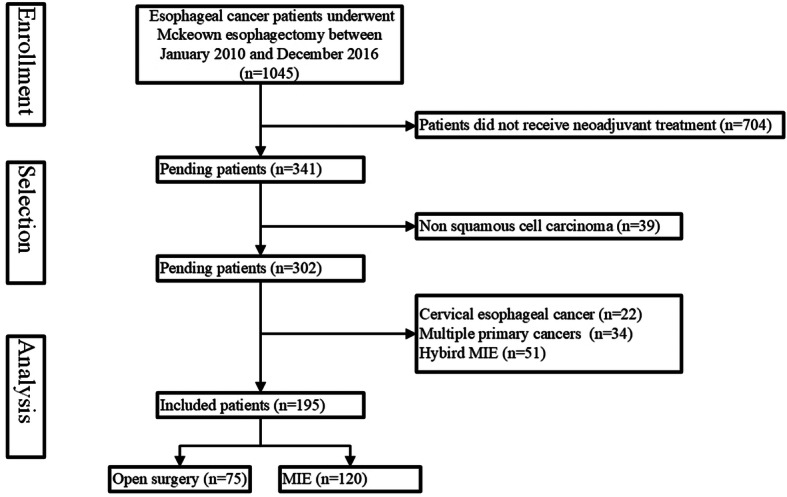
Flow diagram of patient enrollment

For the purpose of this analysis, we retrospectively studied ESCC patients who had received neoadjuvant therapy and underwent Mckeown esophagectomy between January 2010 and December 2016 in our cancer center. Figure 1 summarized the flowchart of patient selection. A total of 1045 patients underwent Mckeown esophagectomy was initially included. Eligible participants were histologically proven squamous cell carcinoma by preoperative biopsy. We excluded patients who did not underwent neoadjuvant treatment. Patients who had cervical esophageal cancer or another malignancy were also excluded. Finally, 195 patients were included for our study. All patients in our cohort received similar regimen of neoadjuvant treatment. For patients received nCT, chemotherapy was 2–3 cycles of cisplatin and vinorelbine, or cisplatin and docetaxel in the preoperative setting; for patients underwent nCRT, preoperative chemotherapy was implemented, with the concurrent radiotherapy (40.0 Gy in 20 fractions for 5 days per week) followed by three incisions esophagectomy. All patients underwent routine examination preoperatively, including upper gastrointestinal endoscopy, barium esophagram, contrast-enhanced computed tomography (CT) scan of the chest and upper abdomen, pulmonary function, and blood sampling.

### Surgical procedures

After 6–8 weeks, neoadjuvant treatment was followed by open or minimally invasive Mckeown esophagectomy. The decision of operative method (MIE or thoracotomy) mostly depends on the discretion of the surgeons. Surgical techniques have been described previously in details [11, 14]. Briefly, open Mckeown esophagectomy involved a right posterolateral thoracotomy in the lateral decubitus position with double tracheal intubation and lung block, upper midline laparotomy, and cervical incision. Initially, a posterolateral incision was made to mobilize the esophagus from the thoracic inlet to the esophageal hiatus and to perform the systemic mediastinal lymphadenectomy. Then, the patient was turned into the supine position and an incision was made in the upper abdomen to mobilized the stomach and to construct the tubularized stomach. Finally, we performed a left cervical incision along the leading edge of the left sternocleidomastoid for the anastomosis using a circular stapler. MIE was firstly performed through a right thoracoscopy in the left lateral decubitus position with four thoracoscopic ports. The thoracic esophagus is mobilized from the thoracic inlet to the diaphragmatic reflection with dissection of the recurrent laryngeal nerve, paraesophageal and subcarinal lymph nodes. After closing the thoracic ports, the patient would be turned to the supine position and the pneumoperitoneum was established. The stomach was mobilized and the abdomen lymph nodes were dissected with five abdominal ports. Then a gastric tube was constructed by preserving the right gastric vessels. Next, a neck incision was made and the esophageal specimen was pull out of the incision. Finally, the specimen was resected and an anastomosis was performed between the cervical esophagus and gastric tube using a circular stapler. Two-field lymphadenectomy was routinely performed in both procedures. The cervical lymph node dissection was only carried out in patients who were detected the suspicious cervical lymph node metastasis preoperatively. Perioperative data including operation time, blood loss, postoperative hospital stay, complications, and in-hospital mortality were collected and analyzed. All patients were recommended to reexamination regularly after being discharge from the hospital, and the follow-up telephone survey was carried out every half year until death or March 2020. The mean follow-up period was 39 months (range 1–120 months). End points of current study were overall survival (OS) and disease-free survival (DFS). OS was defined as the time from the date of diagnosis to the date of death or the last follow-up. DFS was calculated from the date of radical resection to the date of tumor recurrence or metastasis.

### Statistical analysis

SPSS 20.0 software (IBM, Armonk, NY) and GraphPad Prism version 6.0 (GraphPad Software, La Jolla, CA, USA) were applied to perform statistical analysis. Independent sample *t* test and Mann-Whitney *U* test were used to compare continuous variables, while the chi-square test was used for categorical variables. In survival analysis, OS and DFS were calculated by the Kaplan-Meier and then compared by the log-rank test. The univariate and multivariable Cox proportional hazard regression models were served to calculate the hazard ratios (HRs) and 95% confidence intervals (95% CIs). *P* < 0.05 was considered significant.

## Results

### Demographic characteristics

We included 195 ESCC patients underwent neoadjuvant therapy followed by surgery between 2010 and 2016. Neoadjuvant chemotherapy was the primary treatment for 77 patients, and 118 patients underwent neoadjuvant chemoradiotherapy. All patients received Mckeown esophagectomy, of whom 75 underwent open surgery while 120 patients underwent minimally invasive esophagectomy, and patients were grouped by the surgical procedure. The baseline clinical characteristics of the two groups were summarized in Table 1. As the Table 1 showed, all patients received radical resection and with negative margins (R0). The demographic and clinical characteristics of the two groups were no significant differences at baseline, except the operation date, the number of resected lymph nodes and preoperative treatment. The number of patients who underwent MIE was increased after 2014 in our cancer center. Open surgery group harvested more total lymph nodes than MIE group (30.44 ± 15.00 vs 23.53 ± 10.15; *P* < 0.001). After receiving nCRT, the number of patients underwent minimally invasive surgery was greater than the number of those underwent thoracotomy (90 vs 28). Additionally, pathological T stage and differentiation were significantly different between two approaches. However, the nodal stage and ypTNM stage were no differences at baseline (Table 2). Furthermore, seventy-three of 195 patients (37.4%) achieved a complete pathological response (pCR). Eighty-seven patients (44.6%) achieved primary tumoral clearance, and patients who were node-positive prior to neoadjuvant therapy, 126 (64.6%) achieved nodal clearance.

**Table 1:** Baseline characteristics of patients stratified for the surgical approach

	Mckeown esophagectomy		
Demographics	Open surgery	MIE	*P*
**Number (n)**	75	120	
**Age (y)**	58.19 ± 7.82	56.76 ± 6.48	0.088
**Sex**			
Female	10 (13.3)	26 (21.7)	0.185
Male	65 (86.7)	94 (78.3)	
**Pretherapeutic clinical stage**			0.862
I	9 (12.0)	11 (9.2)	
II	19 (25.3)	27 (22.5)	
III	29 (38.7)	50 (41.7)	
IV	18 (24.0)	32 (26.7)	
**Neoadjuvant therapy**			< 0.001
Chemotherapy	47 (62.7)	30 (25.0)	
Chemoradiation	28 (37.3)	90 (75.0)	
**Operation date**			0.003
2010.1–2013.12	45 (60.0)	45 (37.5)	
2014.1–2016.12	30 (40.0)	75 (62.5)	
**R0 resection**	75 (100.0)	120 (100.0)	1.000
**Location**			0.077
Upper third	31 (41.3)	31 (25.8)	
Middle third	29 (38.7)	59 (49.2)	
Lower third	15 (20.0)	30 (25.0)	
**Total resected LNs**	30.44 ± 15.00	23.53 ± 10.15	< 0.001
**Postoperative therapy**			
No	48 (64.0)	93 (77.5)	0.059
Yes	27 (36.0)	27 (22.5)	

Data are mean ± SD or n (%)

**Table 2:** Pathological stage after neoadjuvant treatment of patients stratified for the surgical approach

	Mckeown esophagectomy		
Demographics	Open surgery	MIE	*P*
**Pathological T stage**			< 0.001
0	20 (26.7)	67 (55.8)	
T1	7 (9.3)	12 (10.0)	
T2	14 (18.7)	17 (14.2)	
T3	34 (45.3)	24 (20.0)	
**Pathological N stage**			0.844
N0	47 (62.7)	79 (65.8)	
N1	16 (21.3)	27 (22.5)	
N2	8 (10.7)	10 (8.3)	
N3	4 (5.3)	4 (3.3)	
**Grade**			0.002
Posttreatment change	21 (28.0)	65 (54.2)	
G1	8 (10.7)	4 (3.3)	
G2	28 (37.3)	29 (24.2)	
G3	18 (24.0)	22 (18.3)	
**ypTNM stage**			0.161
I	33 (44.0)	68 (56.7)	
II	14 (18.7)	11 (9.2)	
III	24 (32.0)	37 (30.8)	
IV	4 (5.3)	4 (3.3)	

Data are n (%)

G1 = well differentiated; G2 = moderately differentiated; G3 = poorly differentiated

### Intraoperative factors and perioperative outcomes

As presented in Table 3, patients in the open esophagectomy group suffered from longer operative time and more intraoperative blood loss than patients in the MIE group (390 min vs 330 min, *P* = 0.001; 204 ml vs 167 ml, *P* = 0.021). In addition, the mean hospital stays of the open surgery group was longer than that of the MIE group, however, the difference was not statistically significant (34 days vs 23 days; *P* = 0.068). Furthermore, we discussed the occurrence of major complications after esophagectomy. The most common post-operative complication was anastomotic leakage (9.7% of cases). Anastomotic leakage increased in patients who underwent open esophagectomy (20.0% vs 3.3%, *P* < 0.001, for thoracotomy and MIE, respectively), while the incidence of other postoperative complications did not differ significantly. Two patients underwent reoperation for hemothorax in the minimal invasive group, while no patients underwent reoperation after open esophagectomy. During hospitalization, 4 patients died from acute respiratory distress syndrome (ARDS) after anastomotic leakage, but no differences were found between the two groups (2 vs 2, for thoracotomy and MIE, respectively).

**Table 3:** Comparison of postoperative consequences between the open surgery and minimal invasive approaches

	Mckeown esophagectomy		
Variables	Open surgery	MIE	*P*
**Operative time (min)**	390.93 ± 154.56	330.67 ± 91.96	0.001
**Blood loss (ml)**	204.00 ± 105.83	167.23 ± 107.44	0.021
**Hospital stays (D)**	34.87 ± 61.13	23.82 ± 19.50	0.068
**In-hospital mortality, n(%)**	2 (2.7)	2 (1.7)	0.639
**Complications**			
Anastomotic leakage	15 (20.0)	4 (3.3)	< 0.001
ARDS	2 (2.7)	4 (3.3)	1.000
Pneumonia	1 (1.3)	1 (0.8)	1.000
Chylothorax	0	2 (1.7)	0.549
Reoperation for hemothorax	0	2 (1.7)	0.549
Arrhythmia	1 (1.3)	0	0.348
**Recurrence, n(%)**	34 (45.3)	29 (24.2)	0.003

Data are mean ± SD or n (%)

*MIE* minimally invasive esophagectomy, *ARDS* acute respiratory distress syndrome

### Long-term outcomes

**Fig. 2:**
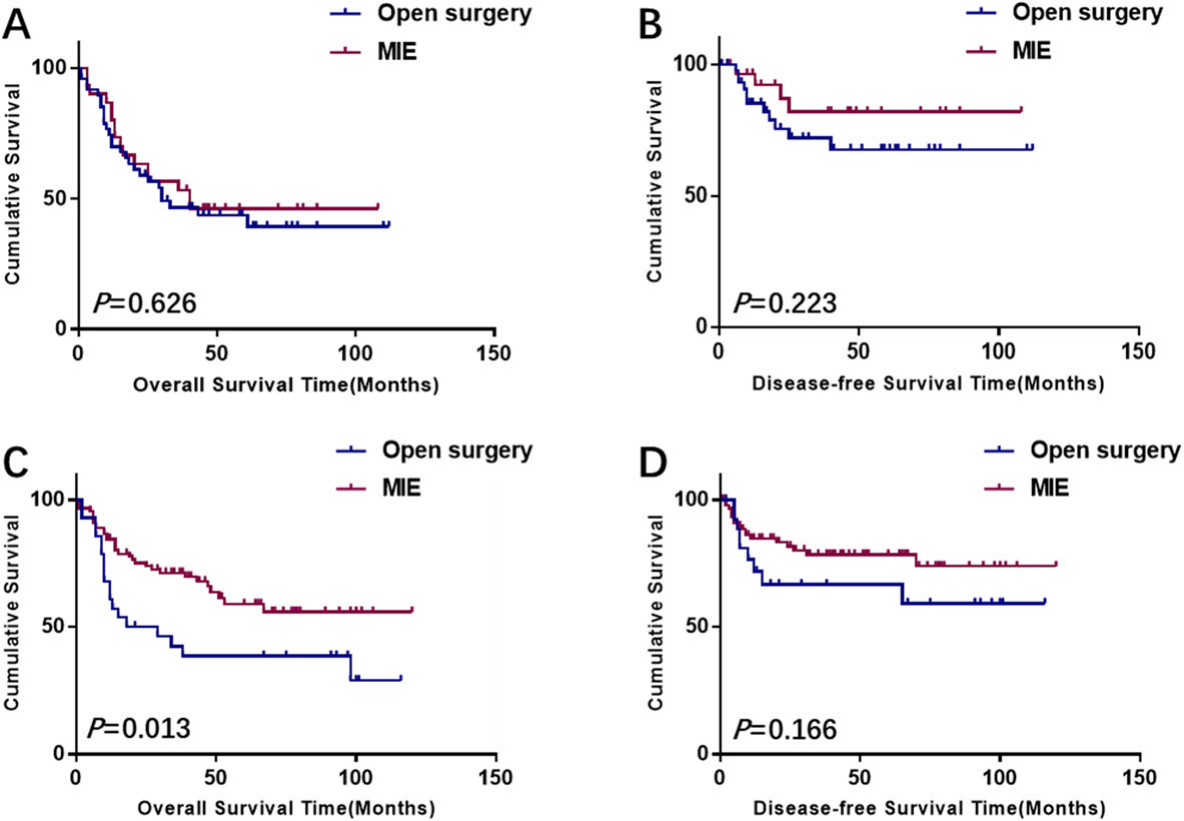
For patients received neoadjuvant chemotherapy, no significant difference was shown in overall survival (log rank = 0.238; *P* = 0.626) (**a**) and disease-free survival (log rank = 1.484; *P* = 0.223) (**b**) between patients who underwent open surgery and minimal invasive procedure. For patients received neoadjuvant chemoradiation, patients in MIE group had significant better overall survival (log rank = 6.197; *P* = 0.013) (**c**) than those in the open esophagectomy group, but no difference regarding disease-free survival (log rank = 1.916; *P* = 0.166) (**d**)

At the end of the follow-up period, a total of 66 patients had died (29 in the thoracotomy group and 37 in the MIE group). 34 patients in the thoracotomy group and 29 patients in the minimal invasive group developed recurrences during follow-up. Considering the effect of different pre-operative treatment on prognosis, we analyzed the survival between two surgical procedures stratified by neoadjuvant therapy. For patients received nCT, the 5-year cumulative survival rates were 39.0 and 45.0% for the open esophagectomy and MIE, respectively. Additionally, the 5-year cumulative survival rates for patients received nCRT were 38.0 and 56.0% for traditional thoracotomy and MIE, respectively. The survival outcomes of the two surgical approaches were presented in Fig. 2. Kaplan-Meier analyses suggested that patients underwent neoadjuvant chemotherapy had no significant differences in OS and in DFS (log rank = 0.238; *P* = 0.626 for OS and log rank = 1.484; *P* = 0.223 for DFS) between the two different surgical methods (Fig. 2a and b). However, for the patients underwent neoadjuvant chemoradiation, there was significant difference in OS between the two procedures (log rank = 6.197; *P* = 0.013), but not in DFS (log rank = 1.916; *P* = 0.166) (Fig. 2c and d).

Cox proportional hazards model demonstrated that the MIE procedure had significant impact on OS in univariate analysis (HR = 0.557; 95% CI, 0.358–0.867; *P* = 0.010) (Table 4). After adjusting potential confounders, minimal invasive surgery was found to be an independently prognostic factor for OS (HR = 0.606; 95% CI, 0.384–0.958; *P* = 0.032) (Table 5). Additionally, the date of surgery was not demonstrated to be related with prognosis. However, sex, T stage, N stage, differentiation, ypTNM, and adjuvant therapy were significantly associated with the prognosis in univariable analysis (all *P* < 0.05) (Table 4). Furthermore, higher ypTNM stage was revealed to be an independent risk factor for OS (*P* = 0.005 for stage II and *P* = 0.006 for stage III). In contrast, receiving postoperative therapy was independently related to a favorable DFS (*P* < 0.001) (Table 5).

**Table 4:** Univariate cox regression analysis of prognostic factors influencing overall survival and disease-free survival

Variables	Overall survival			Disease-free survival		
	HR	95% CI	*P*	HR	95% CI	*P*
**Age(y)**						
≤ 55	1			1		
> 55	0.987	0.629–1.548	0.954	0.703	0.383–1.289	0.254
**Sex**						
Female	1			1		
Male	2.275	1.135–4.559	0.021	2.089	0.820–5.320	0.122
**Surgical approach**						
Open surgery	1			1		
MIE	0.557	0.358–0.867	0.010	0.618	0.337–1.134	0.120
**Operation date**						
2010.1–2013.12	1			1		
2014.1–2016.12	0.891	0.587–1.352	0.588	0.954	0.513–1.771	0.880
**Location**						
Upper third	1			1		
Middle third	0.972	0.581–1.608	0.915	2.083	1.007–4.307	0.048
Lower third	1.134	0.632–2.032	0.674	0.726	0.248–2.124	0.558
**Pathological T stage**						
0	1			1		
T1	2.367	1.181–4.743	0.015	1.219	0.354–4.202	0.754
T2	1.401	0.742–2.646	0.298	1.999	0.907–4.410	0.086
T3	2.531	1.559–4.110	< 0.001	1.584	0.760–3.303	0.220
**Pathological N stage**						
N0	1			1		
N1	1.347	0.806–2.250	0.255	1.207	0.582–2.505	0.613
N2	3.673	2.077–6.496	< 0.001	1.651	0.574–4.751	0.353
N3	4.051	1.822–9.008	0.001	2.060	0.487–8.710	0.326
**Grade**						
Posttreatment change	1			1		
G1	1.738	0.763–3.958	0.188	0.889	0.205–3.850	0.875
G2	1.800	1.078–3.007	0.025	1.181	0.552–2.526	0.668
G3	2.473	1.457–4.196	0.001	2.038	0.970–4.281	0.060
**ypTNM stage**						
I	1			1		
II	1.431	0.699–2.930	0.327	1.618	0.680–3.851	0.277
III	2.238	1.369–3.660	0.001	1.457	0.130–2.910	0.286
IV	3.906	1.505–10.135	0.005	2.296	0.533–9.893	0.266
**Neoadjuvant therapy**						
Chemotherapy	1			1		
Chemoradiation	0.732	0.470–1.141	0.168	1.160	0.617–2.182	0.645
**Postoperative therapy**						
No	1			1		
Yes	0.503	0.319–0.794	0.003	0.111	0.056–0.218	< 0.001

*HR* hazard ratio; *CI* confidence interval; *MIE* minimally invasive esophagectomy. *G1* well differentiated; *G2* moderately differentiated; *G3* poorly differentiated

**Table 5:** Multivariate cox regression analysis of prognostic factors associated with overall survival and disease-free survival

Variables	Overall survival			Disease-free survival		
	HR	95% CI	*P*	HR	95% CI	*P*
**Sex**						
Female	1			–		
Male	2.010	0.994–4.065	0.052	–		
**Surgical approach**						
Open surgery	1			1		
MIE	0.606	0.384–0.958	0.032	0.848	0.449–1.600	0.610
**Location**						
Upper third	–			1		
Middle third	–			2.179	1.026–4.628	0.043
Lower third	–			0.835	0.272–2.564	0.752
**ypTNM stage**						
I	1			1		
II	1.267	0.617–2.600	0.519	1.245	0.515–3.013	0.626
III	2.080	1.254–3.451	0.005	0.918	0.444–1.899	0.817
IV	3.969	1.500–10.505	0.006	1.051	0.236–4.680	0.948
**Postoperative therapy**						
No	1			1		
Yes	0.668	0.415–1.074	0.096	0.115	0.057–0.231	< 0.001

*HR* hazard ratio; *CI* confidence interval; *MIE* minimally invasive esophagectomy

## Discussion

In the current study, we sought to evaluate the impact of surgical methods on short-and long-term outcomes after neoadjuvant therapy in patients with ESCC. We only included patients underwent neoadjuvant treatment followed by Mckeown esophagectomy to guarantee the negative margins and systematic lymphadenectomy. MIE resulted in a shorter operative time, and less bleeding during operation, and after surgery, the incidence of anastomotic leakage was lower than open esophagectomy. For long-term survival, the OS and DFS between the two procedures was comparable in patients underwent preoperative chemotherapy. Nevertheless, MIE was significantly associated with better OS in patients underwent neoadjuvant chemoradiation. Minimal invasive surgery was further demonstrated as an independently prognostic factor for favorable OS.

Traditionally, surgery is considered as the primary treatment for resectable esophageal cancer patients, but the surgical outcomes for the locally advanced disease seems hard to improve. Since previous studies have demonstrated the positive role of neoadjuvant therapy on the prognosis of esophageal cancer patients [3, 6, 15], the use of preoperative therapy followed by surgery has become common practice for the locally advanced ESCC patients in the clinical application. Neoadjuvant CT and CRT contribute to the clearance of micrometastatic disease and tumor down staging, which benefits a more radical surgical resection and a better survival. But at the same time, both chemotherapy and radiotherapy may result in tissue edema and adhesion, and increase the difficulty of the surgical procedure. Furthermore, chemoradiotherapy followed by surgery is associated with significant side effects, including radiation pneumonitis, postoperative pulmonary complications, and pericarditis [16]. Recent years, two primary methods for esophagectomy are traditional thoracotomy and minimally invasive surgery combined thoracoscopy and laparoscopy. Although a number of institutions have investigated the benefits and disadvantages of the two procedures, there is very limited data on the short-and long-term clinical outcomes of the open esophagectomy and MIE concerning ESCC patients underwent neoadjuvant therapy [10, 17–19]. Considering the treatment-related adverse effects, therefore, it is essential to clarify the appropriate surgical method for esophagectomy after induced therapy.

In terms of operative outcomes, our study indicated that shorter operation time and less intraoperative blood loss were noted in the MIE group, which was consistent with the previous reports [19, 20]. However, we found the lymph node yield in open surgery group was higher than in MIE group (mean: 30 vs 23). There is reason to suspect the traditional open surgery is beneficial to perform the systematic lymphadenectomy under direct vision, while the operation field observed by the monitor in MIE was lacking in partial depth perception due to its two dimensions. Although the number of lymph node harvested was fewer in MIE than open surgery in our study, the lymph node yield was in excess of the recommended threshold of 11–15 nodes required for accurate staging [21]. It is well established that dissected lymph node comprehensively provides useful prognostic information after surgery. However, it is controversial whether excise all involved lymph nodes actually improves long-term prognosis [22]. Particularly, the number of lymph nodes removed has relatively low sensitivity for N staging after neoadjuvant therapy. The radical mediastinal lymphadenectomy would prolong the surgery time and the duration of collapsed lung, and increase the risk of damage in nerve and lymphatics. In addition, during surgery, the dissection of the lymph nodes located deep in the mediastinum is inevitable to avoid stretching or compressing of the lung parenchyma, which is related to the pulmonary complications after esophagectomy. Furthermore, lung toxicities are the most common adverse effect of neoadjuvant chemoradiotherapy, and the incidence of postoperative pulmonary complications was demonstrated a significant increase in patients received neoadjuvant treatment [16, 23]. Thus, the significance of extensive lymphadenectomy after induction needs to be further discussed.

In most previous studies, the leakage rate was reported as similar for both procedures [10, 19, 24]. Nevertheless, in this study, the major complications between two groups were comparable except the anastomotic leakage. Open esophagectomy was suggested to be associated with a higher risk of cervical anastomotic leakage than MIE. According to our clinical experience, postoperative pain in patients underwent open esophagectomy is still more severe than those had MIE, even if the high doses of analgesic has been taken in the first week after operation. The intense pain would lead to the fragile cough and patients are unwilling to expectorate spontaneously. Meanwhile, the intense pain prevents patients from getting out of bed and do activities as soon as possible. These disadvantages may cause pulmonary infection and hypoxemia, which are the major risk factors for anastomotic leakage.

The comparisons of long-term outcomes between open esophagectomy and MIE have been widely discussed [17, 19, 25, 26]. However, the effect of different procedures on prognosis is still controversial. Most studies demonstrated that MIE appeared to produce better survival compared with open surgery [19, 26], while similar outcomes between two procedures were also reported [17, 25, 27]. In our study, Cox model demonstrated that MIE was an independently prognostic factor (HR = 0.606; 95% CI, 0.384–0.958; *P* = 0.032). Nevertheless, in the subgroup analysis, we found the prognostic effect of surgical method was various in patients received different neoadjuvant treatments. For patients underwent nCT, OS and DFS were comparable between patients in open and MIE group. On the contrary, significant difference in OS between the two surgical methods was observed in patients underwent nCRT and MIE related to a better prognosis. The reason may be concluded as follows: firstly, neoadjuvant chemoradiation improves the pCR rate compared with chemotherapy alone, which shrinks the tumors dramatically [28]. In particular, MIE with the thoracoscope and laparoscope could amplify the surgical filed, which is beneficial for the dissection of tumor in relatively small size and decreases the surgery duration. Thus, patients with nCRT followed by minimal invasive surgery demonstrated improved OS. Subsequently, some ESCC patients could not tolerate the concurrent preoperative chemoradiotherapy due to the serious side effects and only underwent neoadjuvant chemotherapy. Under the circumstances, pCR is hardly achieved and the size of primary tumor would not change as obviously as after nCRT. According to our experience, MIE did not prevail over traditional open surgery in dissecting complex structure or oversize tumor surrounded by denser tissue. Open esophagectomy with hand-assisted seems more precise in operating complicated surgery than MIE that can only rely on the surgical apparatus. Number of harvested nodes was less in the MIE group, suggesting that MIE may involve a steep learning curve. Therefore, comparing MIE with the open procedure to esophageal resection showed comparable long-term survival in patients received nCT.

In this study, we only enrolled ESCC patients who underwent neoadjuvant therapy followed by three incisions esophagectomy with traditional open surgery and total MIE. Other approaches such as Ivor Lewis esophagectomy or hybrid MIE [26] were excluded, which guaranteed the en bloc resection and normalized lymphadenectomy. Nevertheless, it is important to recognize the limitations of our study. This is a retrospective analysis, and the selection bias is inevitable. Patients were not randomly assigned to open esophagectomy or MIE group but were treated based on surgeon evaluation and patient deliberation. Therefore, patients in advanced disease would be inclined in the open esophagectomy group, which may lead to the worse outcomes than the other group intrinsically. In addition, the sample size is limited because of the rigorous enrollment criteria, and large population study is needed to validate the association between surgical procedures and prognosis for ESCC patients underwent neoadjuvant treatment.

## Conclusion

Preoperative chemotherapy or chemoradiotherapy followed by esophagectomy has produced encouraging results for ESCC patients. The current study confirms that minimally invasive Mckeown esophagectomy is safe and feasible for ESCC patients who underwent neoadjuvant therapy. MIE approach presented less blood loss, shorter surgery period, and lower rate of anastomotic leakage than open esophagectomy. The effect of two surgical procedures on long-term oncological results was various, depending on the scheme of neoadjuvant treatment. For patients underwent neoadjuvant chemotherapy, OS was comparable between MIE and open surgery. However, MIE was associated with better prognosis in patients received neoadjuvant chemoradiation. Randomized controlled researches with large sample size are needed to further validate our results.

## Data Availability

The datasets analyzed during this study is available from the corresponding author on reasonable request.

## Abbreviations

MIE: Minimally invasive esophagectomy
ESCC: Esophageal squamous cell carcinoma
OS: Overall survival
DFS: Disease-free survival
EC: Esophageal cancer
nCRT: Neoadjuvant chemoradiotherapy
nCT: Neoadjuvant chemotherapy
CT: Computed tomography
HRs: Hazard ratios
CI: Confidence interval
ARDS: Acute respiratory distress syndrome
pCR: pathologic complete response
